# Exploring Cross-Cultural Sensory Acceptance of Vinegar-Based Dipping Sauces: A Taiwanese Consumer Study with Dumplings

**DOI:** 10.3390/foods14132398

**Published:** 2025-07-07

**Authors:** Jung-Kuei Ker, Ming-Chen Chiang, Ching-Sung Lee, Yen-Cheng Chen

**Affiliations:** 1 Ph.D. Program in Nutrition and Food Science, College of Human Ecology, Fu Jen Catholic University, New Taipei City 242062, Taiwan; 2 Department of Food Beverage Management, Mackay Junior College of Medicine, Nursing and Management, Taipei 112021, Taiwan; 3 Department of Restaurant, Hotel and Institutional Management, College of Human Ecology, Fu Jen Catholic University, New Taipei City 242062, Taiwan; 4 Department of Applied Science of Living, College of Agriculture, Chinese Culture University, Taipei 11114, Taiwan

**Keywords:** cross-cultural sensory perception, vinegar, flavor acceptance, Taiwanese cuisine, multisensory experience, culinary adaptation, food culture

## Abstract

Vinegar functions not only as a sensory enhancer but also as a culturally embedded culinary element across global food systems. In Taiwanese cuisine, black vinegar represents a traditional staple, particularly associated with dumpling consumption, whereas Italian balsamic vinegar is renowned for its aromatic complexity and nuanced sweetness, highly esteemed in Western gastronomy. Despite their culinary significance, limited empirical research has examined how these culturally distinct condiments are perceived when applied beyond their traditional contexts, especially in iconic national dishes. This study investigates Taiwanese consumers’ cross-cultural sensory responses to dumplings paired with either local black vinegar or imported balsamic vinegar. Through a structured sensory evaluation encompassing appearance, aroma, taste, and overall impression, this research explores how sensory cues and cultural expectations interact to shape flavor preferences. The results indicate that although visual attributes were rated similarly, balsamic vinegar’s distinctive aroma and taste elicited significantly greater sensory engagement, suggesting a latent openness to reinterpretation and hybridization within established food practices. These findings were supported by one-way ANOVA results, which revealed significant differences among the three groups for aroma (F = 6.30, *p* < 0.01), taste (F = 7.21, *p* < 0.01), and overall evaluation (F = 15.15, *p* < 0.001). By integrating sensory analysis with cultural food studies, this research advances the understanding of how multisensory cues influence consumer acceptance across cultural contexts. It further highlights the dynamic interplay between cultural familiarity and sensory novelty in flavor perception. These insights yield practical implications for culinary innovation, global flavor localization, and the development of culturally responsive food products.

## 1. Introduction

Vinegar is a natural food product produced through a fermentation process, which includes alcoholic fermentation followed by acetic acid fermentation. This process involves the use of raw materials rich in carbohydrates, such as grapes, apples, rice, and other carbohydrate-rich ingredients. A large number of studies have demonstrated that the components of edible vinegar are beneficial to health and possess multiple functional properties, including antibacterial, anti-infective, antioxidant, anticancer, blood sugar control, lipid metabolism regulation, and weight loss [1,2,3,4]. Currently, vinegar can be mainly divided into two categories based on its raw materials: grain vinegar and fruit vinegar [5].

Vinegar has long been regarded as an important culinary ingredient in many Asian countries [6,7,8]. It can be used both as a condiment for foods and as an ingredient in dishes, as it can alter people’s overall preference for a particular food or meal [9]. Moreover, adding vinegar to the diet can improve health conditions [6,10]. Vinegar is rich in nutrients and bioactive components, including amino acids, sugars, organic acids, polyphenols, melanoidins, and tetramethylpyrazine [4,10,11]. These components can help maintain acid/base balance, regulate cellular metabolism, provide energy, and enhance the immune system [1,12]. Grain vinegar is very common in Asian countries, with most Asian vinegars made from rice, but other grains such as millet, sorghum, barley, and husks are also used [10]. It is evident that the addition of vinegar to the diet is highly beneficial to human health.

In Taiwan, many people are very fond of using black vinegar as a condiment or dipping sauce. When food is combined with seasonings, it can alter the sensory attributes of the food, including its color, texture, and aroma, resulting in different sensory effects [13]. Therefore, when vinegar is mixed with food, its slightly sour flavor can enrich the taste experience by enhancing the aroma, adding a tangy sharpness to the taste, and subtly altering the overall mouthfeel of the dish. These sensory changes can make the food more appetizing, refreshing, and memorable, while also introducing richer layers of flavor to the overall profile. The commonly used black vinegar in Taiwan is a traditional rice vinegar [14], which is typically made from a base of rice, wheat, millet, and sorghum, combined with various vegetables and fruits such as onions, orange juice, carrot juice, tomatoes, fennel, and celery. Its color is typically black.

In contrast, in European countries, high-quality balsamic vinegar is preferred by many users. Balsamic vinegar originates from the Modena region of Italy and is renowned for its rich aroma and balanced sweet and sour taste, making it highly popular in Western cuisine [15]. When applied to various types of foods, it produces a unique flavor, especially when added directly to dishes, creating a distinctive taste experience. According to previous research [3], authentic balsamic vinegar contains various trace elements and bioactive compounds such as acetic acid, polyphenols, melanoidins, ligustrazine, tryptophol, and organic acids. These components contribute to its distinctive characteristics and widespread use as a popular household condiment in Italy. The distinctive characteristics of balsamic vinegar have earned it high acclaim in the international market, where it is increasingly favored as a condiment [16], becoming an indispensable seasoning in kitchens worldwide [17].

Previous studies on sensory evaluation have primarily focused on assessing the taste, aroma, and overall acceptability of various foods and beverages to determine consumer preferences—namely, whether users liked or disliked a product [18,19,20]. Consequently, many of these studies have developed diverse scales to measure the degree of preference [18]. Within this context, a growing body of research has explored cross-cultural aspects of food perception, offering valuable insights into how consumer behavior varies across different countries and cultural settings [19,20,21]. Specifically regarding vinegar, only a limited number of studies have conducted systematic sensory evaluations, particularly in cross-cultural contexts. For instance, Torri et al. [20] compared Korean and Italian consumers’ perceptions of balsamic vinegar, highlighting notable cultural differences in aroma and flavor evaluation. Chen et al. [22] have further emphasized the need to investigate how sensory preferences for vinegar vary across countries and regions, calling for expanded vinegar-focused sensory research to support product development and localization strategies. These findings underscore the importance of incorporating vinegar into food choice and sensory evaluation research. Understanding how consumers perceive and accept different types of vinegar can provide a scientific basis for culinary innovation and support the development of culturally adaptive flavor strategies [17,22]. It can be applied to various types of foods, especially in the evaluation of fermented products, and it enables researchers to understand consumers’ taste preferences and dietary habits across different regions and cultural backgrounds [17,22,23,24]. Through sensory evaluation methods, it is possible to assess the popularity of different foods among different groups and further explore the cultural and social factors underlying these preferences [25]. This approach is of significant importance to market development and product positioning in the food industry while also contributing to the promotion of cross-cultural exchange and understanding [20].

In addition, consumers tend to exhibit cautious behavior when evaluating the sensory attributes of foods, such as color, texture, and flavor. They are sometimes capable of detecting lower levels of odors and other sensory characteristics than instruments can, and unlike analytical devices, humans can assess the hedonic value of food and predict preferences. Through consumer-based experiments, the food industry can better determine the overall acceptability and sensory characteristics of food products [26,27]. Regarding taste testing, two key factors influence evaluation outcomes: first, consumers’ ability to perceive differences among products; and second, their overall preference for each product [28]. The recent literature has highlighted both practical applications and methodological considerations in vinegar sensory evaluation. Reinaa et al. [29] conducted a sensory characterization of Argentinean wine vinegars using triangle tests and preference ranking tests. The results demonstrated statistically significant consumer preferences for traditional and rapid fermentation vinegars, suggesting that sensory techniques can effectively differentiate between vinegar types. The study also emphasized that aroma and flavor are among the most critical quality parameters for consumers. Sensory evaluation plays a crucial role in assessing food quality and serves as a valuable complement to broader food research. One widely adopted approach for collecting consumer insights is hedonic sensory testing, which assesses product acceptance and preference. Furthermore, Chen, Ma, Corradini, and Giudici [22] conducted a comprehensive review of sensory evaluation methods applied to Italian Traditional Balsamic Vinegar. Their review stressed the importance of tailoring sensory methodologies to specific evaluation objectives. It also examined multiple facets of vinegar sensory analysis, including descriptor vocabulary, recording methods, evaluation sequence, test environment, psychological bias, panelist training, and sample handling, providing practical recommendations for improving the accuracy and reliability of vinegar sensory assessments.

Despite the clear differences in production origin and sensory profiles, there is limited empirical evidence on how consumers respond to cross-cultural condiment substitutions in traditional meal contexts. This study seeks to address this gap by offering an exploratory analysis of consumer sensory responses to balsamic vinegar as a substitute for black vinegar in Taiwanese dumplings, thereby extending the literature on flavor localization and cross-cultural food integration.

## 2. Materials and Methods

### 2.1. Vinegar Samples

**Figure 1:**
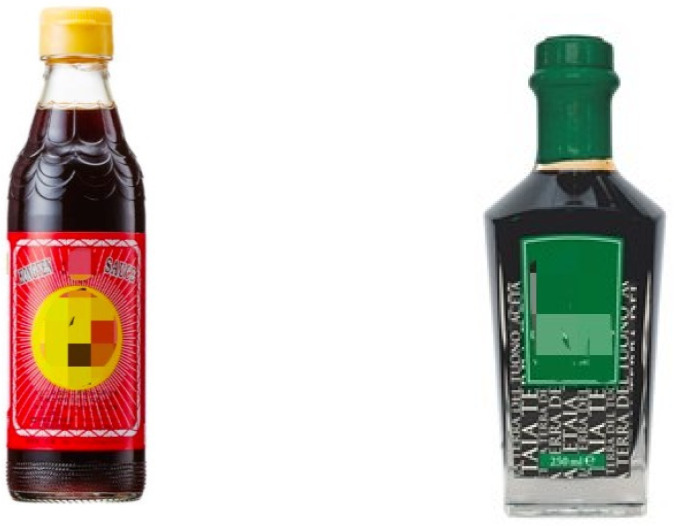
Black vinegar and balsamic vinegar.

**Figure 2:**
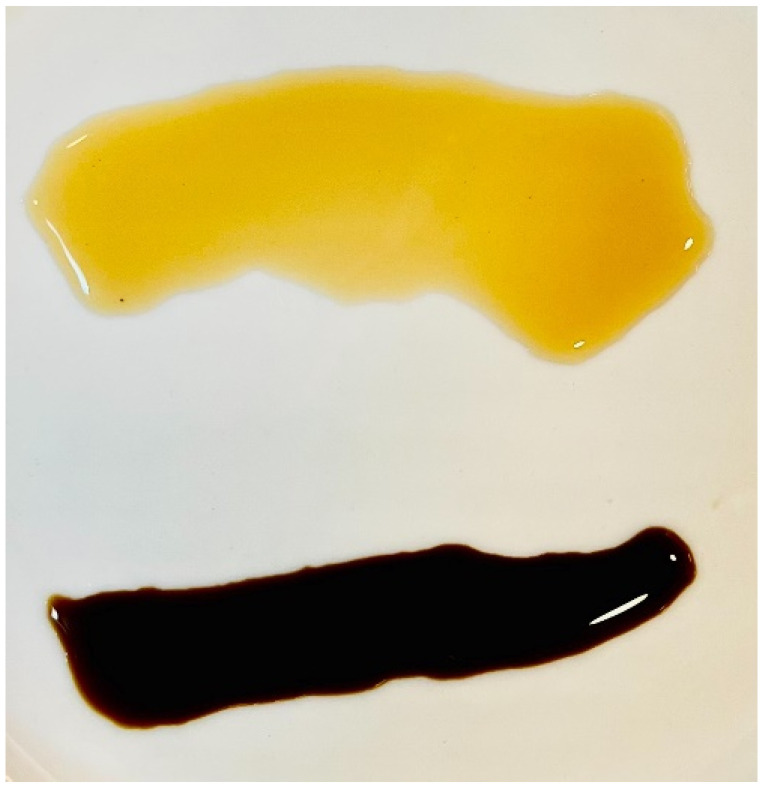
Black vinegar (**Top**) and balsamic vinegar (**Bottom**). Note: Due to its thin consistency and high translucency, black vinegar appears lighter in color when presented in small amounts. In contrast, balsamic vinegar is thicker and less translucent, resulting in a darker appearance.

This study utilized well-known and commonly available black vinegar and balsamic vinegar brands sold in Taiwan as sample products, with acidity levels of 1.8% and 6%, respectively. In the sensory evaluation of Chinese-style dumplings (containing pork and cabbage), each participant was provided with 1 mL of either black vinegar or balsamic vinegar for tasting (as shown in Figure 1 and Figure 2). Most sensory characteristics of food can only be effectively and meaningfully assessed by human participants, as scientific instruments are unable to fully capture the subjective experience of taste and smell. However, to ensure accuracy and consistency, when participants are treated as sensory measurement instruments, all testing procedures and conditions must be strictly standardized to minimize errors caused by psychological factors [30].

In this study, black vinegar, commonly used as a dipping sauce for traditional Chinese dumplings, served as the control condition, while balsamic vinegar, less commonly used in Chinese cuisine, was designated as the experimental condition. To eliminate variations in absorption and dipping behavior that could affect the results, participants were instructed to place the dumpling on a ceramic spoon and pour a fixed amount of vinegar onto it, ensuring that the dumpling was fully immersed in the sauce. This procedure minimized inconsistencies in the amount of vinegar consumed across participants. Moreover, participants were not informed about the specific type of vinegar they were tasting in order to avoid any preconceived expectations that might influence their sensory evaluations.

### 2.2. Dumpling Samples

**Figure 3:**
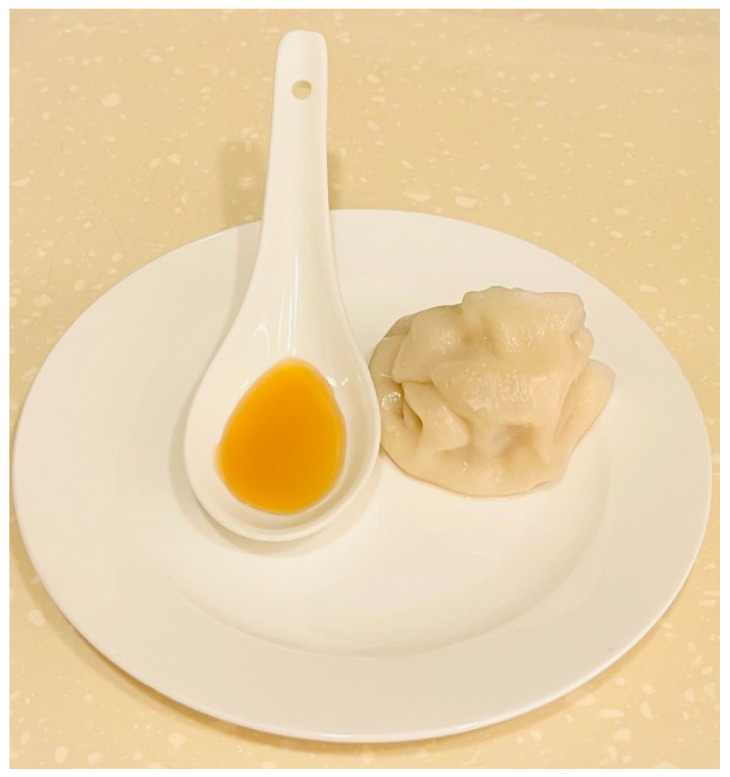
Black vinegar and dumpling samples.

**Figure 4:**
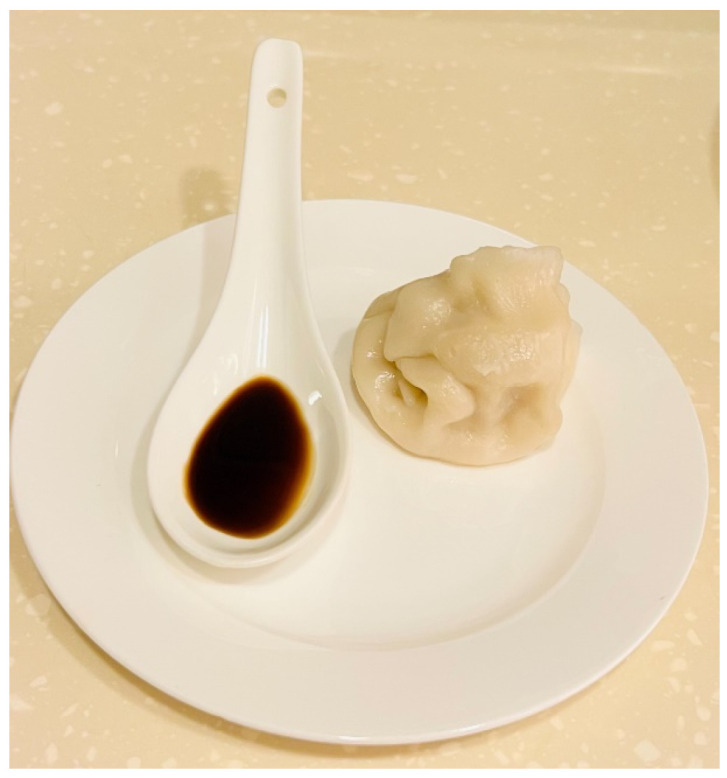
Balsamic vinegar and dumpling samples.

For the sensory evaluation of dumplings by the participants, the experiment utilized 65g dumplings, accompanied by 1 mL of either black vinegar or balsamic vinegar as a dipping sauce (as shown in Figure 3 and Figure 4). The dumplings used in the study were frozen dumplings produced by the well-known Taiwanese company XING JI FOOD, ensuring the consistency of the sample quality. The ingredients of the dumplings included flour, water, pork leg meat, cabbage, salt, sugar, soy sauce, and sesame oil. The cooking process involved placing the frozen dumplings into boiling water for approximately eight minutes. Once the dumplings floated to the surface, they were promptly removed from the pot and plated. Each sample was then served to participants within approximately two minutes to ensure consistent serving conditions and a palatable temperature for sensory evaluation.

### 2.3. Participant Recruitment and Screening

This study recruited participants through Facebook and university bulletin boards, inviting interested consumers to voluntarily complete a registration form. The form included information about the study’s purpose, procedures, and an informed consent statement. Participants were required to confirm their understanding of the procedures and complete a dietary habits survey before proceeding to the next stage of sensory evaluation. Ultimately, a total of 60 consumers were recruited as participants (Table 1 presents the demographic data of the participants).

Participants were required to indicate in the dietary habits survey that they are dumpling consumers who consume dumplings at least once per month to qualify for the study. Additionally, they were asked to confirm that they do not have any major illnesses or conditions that could affect their sense of taste or smell, ensuring their physical health [31]. The survey also collected information about the stores where they purchase dumplings, their preferred flavors, and any known food allergies. All participants received a gift card as compensation after completing the sensory evaluation.

### 2.4. Procedure

This research project has been approved by the Institutional Review Board (IRB) of Mackay Junior College of Medicine, Nursing, and Management (Approval Number: MKC112R12). All participants received a detailed explanation of the experimental procedures and signed a written informed consent form before joining the study. To comply with the experimental requirements, participants were instructed to refrain from smoking, eating, or drinking any beverages other than plain water for at least two hours before entering the sample evaluation area [32]. In the sample evaluation area, the research team installed an air purifier to remove any odors from the air, minimizing interference with participants’ olfactory judgments. Bright white lighting was used to prevent lighting color tones from affecting visual sensory perception. The experimental environment was also soundproofed to ensure a quiet and secure setting. Each participant conducted the sample evaluation individually.

The core objective of this study is to explore whether consumers can accept balsamic vinegar as a new option when paired with traditional Chinese dumplings. To achieve this, we will design a sensory evaluation experiment that examines consumer perceptions of Chinese dumplings paired with different types of vinegar. Sensory evaluations were conducted across four sensory dimensions: visual, olfactory, gustatory, and overall evaluation. The study design divided the dumpling samples into three groups (A1, A2, and B). Group A1 consisted of Chinese dumplings paired with black vinegar, Group A2 also featured Chinese dumplings with black vinegar, and Group B included Chinese dumplings paired with balsamic vinegar. To control for potential biases arising from presentation order and sampling position, a Latin Square Design was employed. This design allocated each sample to different participant groups in a balanced sequence, ensuring that each sample appeared at each evaluation position (e.g., first, second, third) exactly once. This approach ensured balanced and fair sample presentation, effectively enhancing the internal validity of the sensory evaluations and the interpretability of the collected data. The findings of this study were anticipated to provide new directions for innovation and development in the traditional Chinese food market.

The researchers prepared freshly boiled Chinese dumplings on white porcelain plates. After being removed from the pot, the dumplings were quickly plated and served to participants within approximately two minutes, ensuring that the samples were delivered at a palatable temperature and under consistent conditions. To prevent participants from being influenced by the flavors of other samples during the sensory evaluation process, participants were required to rinse their mouths after tasting each type of sample. This rinsing procedure helped eliminate any residual flavors and ensure oral cleanliness. Participants were also asked to complete the sensory evaluation questionnaire after each sample. Subsequently, they proceeded to the next sample, strictly following the prescribed tasting order without making any arbitrary changes. This protocol was designed to maintain the most authentic and rigorous experimental conditions. Each dumpling was paired with exactly 1 mL of vinegar, pre-portioned into a ceramic spoon to ensure consistency across participants. This quantity was carefully selected because both black vinegar and balsamic vinegar have strong and distinctive flavors; larger amounts would have overwhelmed the dumpling’s natural taste and compromised the sensory evaluation’s integrity. The use of a ceramic spoon further controlled for dipping variability; participants were instructed to place the dumpling into the spoon and consume them together.

This study is based on the research findings of Swiader and Marczewska [33] and Ker et al. [17] regarding sensory evaluation factors in visual, olfactory, gustatory, and overall flavor dimensions. Accordingly, the following definitions of sensory evaluation factors were developed:

1. Visual: This refers to the participants’ preference for the appearance of the samples. Specifically, it examines how participants perceive the visual appeal of Chinese dumplings topped with an equal amount of either black vinegar or balsamic vinegar.
2. Olfactory: This dimension pertains to the participants’ perception of the aroma emitted by the sample combinations. In this study, black vinegar, which is fermented from grains, and balsamic vinegar, which is made from grapes, were applied in equal quantities to Chinese dumplings. The sensory evaluation aimed to examine how these distinct aromatic profiles influence participants’ olfactory preferences when paired with the same base food, allowing for a comparison of the perceived fragrance between the two vinegar types.
3. Gustatory: This refers to the participants’ perception of basic taste attributes, such as sourness, sweetness, bitterness, spiciness, saltiness, and umami. The primary objective is to understand participants’ taste preferences for Chinese dumplings seasoned with an equal amount of either black vinegar or balsamic vinegar.
4. Overall Flavor: This refers to the participants’ overall preference for the flavor of the samples. Beyond the sensory evaluations of visual color, olfactory aroma, and gustatory taste, this dimension assesses participants’ overall sensory impressions and acceptance of the flavor of Chinese dumplings when seasoned with an equal amount of either black vinegar or balsamic vinegar.

The questionnaire primarily involves participants completing a sensory evaluation survey after tasting Chinese dumplings seasoned with black vinegar and balsamic vinegar. Participants are asked to rate the sensory attributes of the samples based on four dimensions: visual color, olfactory aroma, gustatory taste, and overall flavor. The evaluation is conducted using a five-point Likert scale, with a total of four sensory evaluation factors: visual color, olfactory aroma, gustatory taste, and overall flavor.

### 2.5. Data Analysis

This study employed SPSS 24.0 for Windows for data analysis. The analytical methods included descriptive statistics, independent samples *t*-tests, and one-way analysis of variance (ANOVA). All statistical tests were conducted with a significance level set at α = 0.05. Given the single-session evaluation design, each participant sequentially evaluated three types of dumpling samples with different dipping sauce conditions (A1, A2, and B). Thus, each sample group received evaluation scores from all 60 participants, which served as the basis for statistical analysis. First, an independent samples *t*-test was conducted to assess the consistency between the two black vinegar samples (A1 and A2) across four sensory dimensions: visual, olfactory, gustatory, and overall evaluation. Subsequently, additional independent samples t-tests were performed to compare the sensory evaluations between the black vinegar samples (A1 and A2) and the balsamic vinegar sample (B). Finally, a one-way ANOVA was applied to examine whether significant differences existed among the three groups across the various sensory attributes.

## 3. Results

### 3.1. Sensory Consistency and Sample Representativeness Analysis of Group AA Using Independent Samples t-Test

The study first verified the consistency of sensory evaluation between the samples of Group A1 and Group A2. As shown in Table 2, the results indicated that there were no significant differences between the two groups in terms of visual perception (t = −1.15, *p* > 0.05), olfactory perception (t = −1.15, *p* > 0.05), gustatory perception (t = −1.15, *p* > 0.05), and overall sensory evaluation (t = −1.15, *p* > 0.05). These findings demonstrate that participants’ evaluations of the two groups of samples did not show significant differences across the four sensory dimensions. Therefore, it can be inferred that both Group A1 and Group A2 were samples using black vinegar as the dipping sauce, and participants demonstrated consistent sensory recognition between the two. This result supports the assumption of homogeneity between the two groups, serving as a foundation for subsequent comparative analysis.

### 3.2. Independent Samples t-Test Results for Sensory Evaluation of Group A1 and Group B

According to the results of the independent sample *t*-test shown in Table 3, there was no significant difference between Group A1 and Group B in terms of “visual perception” (t = −1.35, *p* > 0.05), indicating that the two groups of samples were rated similarly in appearance. However, significant differences were observed in “olfactory perception” (t = −2.69, *p* < 0.01) and “gustatory perception” (t = −2.69, *p* < 0.05), indicating that the two products exhibited distinct sensory perceptions in terms of aroma and taste. Regarding “overall sensory evaluation,” the test results revealed a highly significant difference (t = −4.27, *p* < 0.001), demonstrating that participants’ overall acceptance of the samples in Group B was significantly higher than that of Group A1. In summary, aside from the visual dimension, the olfactory, gustatory, and overall sensory dimensions all exhibited significant differences between the two groups.

### 3.3. Independent Samples t-Test Results for Sensory Evaluation of Group A2 and Group B

According to the results of the independent sample *t*-test shown in Table 4, there was no significant difference between Group A2 and Group B in terms of “visual perception” (t = −1.07, *p* > 0.05), indicating that the two products did not exhibit a clear distinction in appearance. However, significant differences were observed in “olfactory perception” (t = −2.36, *p* < 0.05) and “gustatory perception” (t = −2.84, *p* < 0.01), indicating that the two samples differed in terms of sensory perception related to aroma and taste. Further examination of “overall sensory evaluation” revealed a highly significant difference (t = −4.65, *p* < 0.001), demonstrating that participants’ overall acceptance of the samples in Group B was significantly higher than that of Group A2. In summary, except for the visual dimension, all other sensory dimensions showed significant differences between the two groups.

**Figure 5:**
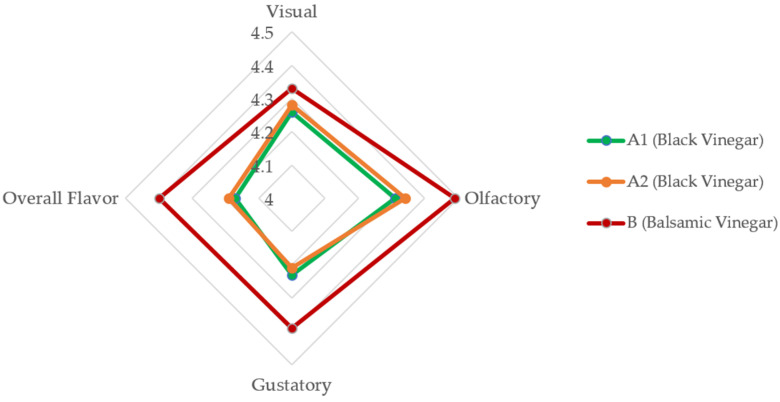
Sensory evaluation radar chart of black vinegar and balsamic vinegar.

From the perspectives of olfactory, gustatory, and overall sensory perception, significant differences were observed between the black vinegar and the balsamic vinegar (as shown in Figure 5). Specifically, balsamic vinegar demonstrated superior performance in terms of aroma complexity, flavor richness, and overall sensory appeal. On the other hand, no significant difference was found in visual perception between the two vinegar options, indicating that the visual appearance of the dipping sauces did not strongly influence participant preferences.

### 3.4. One-Way ANOVA Results for Sensory Evaluation of Group A1, Group A2, and Group B

According to the results of the one-way analysis of variance (ANOVA) presented in Table 5, the three groups of samples (Group A1, Group A2, and Group B) did not show significant differences in “visual perception” (F = 1.53, *p* > 0.05), indicating that the visual evaluation of the samples tended to be consistent across the groups. However, significant differences were observed in “olfactory perception” (F = 6.30, *p* < 0.01) and “gustatory perception” (F = 7.21, *p* < 0.01), demonstrating that the three products were distinguishable in terms of aroma and taste. Moreover, “overall sensory evaluation” displayed a highly significant difference (F = 15.15, *p* < 0.001), further highlighting the differences in overall acceptance among the samples. In summary, the three sample groups exhibited statistically significant differences in olfactory perception, gustatory perception, and overall evaluation.

## 4. Discussion

This study employed sensory evaluation methods to investigate the impact of different dipping sauce samples on consumers’ sensory perceptions, specifically comparing the use of black vinegar (Group A1 and Group A2) and balsamic vinegar (Group B) as dipping sauces for dumplings. First, the results confirmed that there were no significant differences between Group A1 and Group A2 in terms of visual, olfactory, gustatory, and overall sensory evaluations, indicating that these two groups were homogeneous samples. This finding provides a reliable basis for the subsequent experimental design. Second, when comparing the sensory performance between the black vinegar groups (A1 and A2) and the balsamic vinegar group (B), significant differences were found in olfactory perception, gustatory perception, and overall sensory evaluation. Among these, the difference in overall sensory scores was the most pronounced, indicating that consumers exhibited a higher level of acceptance and preference for the flavor and overall mouthfeel of balsamic vinegar. Furthermore, the one-way analysis of variance (ANOVA) also supported the existence of statistically significant differences among the three groups in terms of olfactory, gustatory, and overall sensory dimensions, validating that sensory evaluation effectively distinguished the flavor differences between the samples. While balsamic vinegar is already a well-established commercial product in Western countries, the findings of this study suggest that it holds potential for market acceptance in East Asian culinary contexts, particularly as a dipping sauce for dumplings. In this setting, it may serve as a novel alternative to traditional black vinegar and contribute to expanding the flavor diversity of Chinese cuisine.

### 4.1. Sensory Evaluation of Visual Perception

Both black vinegar and balsamic vinegar share a generally dark color tone in their visual appearance, but they exhibit noticeable differences to a certain extent. Black vinegar typically appears as a clear, highly fluid, dark brown or blackish-brown liquid. Its color is more transparent, and its texture is relatively thin, giving a visually light and delicate impression. In contrast, balsamic vinegar, which undergoes a prolonged aging and concentration process, is usually thicker, more viscous, and displays a saturated dark brown color. Under light, it may even exhibit a slight sheen, presenting a richer and more mature visual appearance. However, when applied as dipping sauces for dumplings, both types of vinegar, being dark-colored liquids, show minimal visible differences on the surface of the food due to the small amount used. As a result, participants found it difficult to distinguish between the two sauces based on visual appearance alone. This finding aligns with the perspective proposed by Spence [34], which suggests that although color and flavor can establish expected connections, when color differences are subtle or consumers are highly familiar with a particular type of food, the impact of visual perception may be replaced by other sensory modalities. Similarly, Zhang, Elsweiler, and Trattner [35] emphasized that visual familiarity is closely related to cultural background and past experiences. When consumers are accustomed to associating dark-colored vinegars with traditional dipping sauces, their recognition mechanisms tend to rely more on olfactory and gustatory cues rather than visual attributes. Furthermore, Torrico et al. [36] pointed out that cultural familiarity reduces sensitivity to color differences and shifts the judgment focus toward more direct flavor experiences. In summary, although balsamic vinegar exhibits slightly higher viscosity and a more intense color depth than black vinegar, their visual impact appears nearly identical in the actual consumption scenario. This similarity in visual presentation led to the absence of significant visual differentiation in sensory evaluation within this study.

### 4.2. Sensory Evaluation of Olfactory Perception

The results of this study indicated that there were significant differences in sensory evaluations of the olfactory dimension among the three sample groups, with balsamic vinegar showing a notably superior aroma performance compared to black vinegar. Balsamic vinegar emitted a rich aroma characterized by ripe fruity notes and a subtle hint of barrel-aged maturation, offering a multi-layered and smooth fragrance that immediately evoked positive emotions and flavor expectations among the participants. In contrast, the aroma of black vinegar was relatively simple, dominated by a singular fermented acidic scent. Although it possessed the recognizable characteristic of traditional flavor, its aromatic profile was more direct and restrained, lacking the multi-dimensional olfactory stimulation found in balsamic vinegar. This finding can be interpreted from two perspectives: sensory psychology and cultural perception. First, according to the study by Hua, Dong, and Maier [37] using an animal model, the association between taste and aroma is established through repeated experiences, and such experiential olfactory/gustatory synergy can stabilize individual preference tendencies. Therefore, when participants were first exposed to balsamic vinegar, its familiar yet novel fruity and mature aroma could easily trigger pleasant sensory memories and positive flavor associations. Furthermore, Spence [38] pointed out that aroma has an “expectation-shaping” function, where olfactory signals begin to influence consumers’ perceptions of a product’s flavor and quality even before the taste experience. In summary, the olfactory superiority of balsamic vinegar is not only due to the complexity and pleasantness of its aroma but is also closely related to participants’ previous sensory experiences and cultural acceptance. These factors collectively contributed to the significant difference in olfactory evaluations between balsamic vinegar and black vinegar.

### 4.3. Sensory Evaluation of Taste Perception

In terms of gustatory evaluation, the three sample groups also demonstrated significant differences, with balsamic vinegar showing the most outstanding performance in terms of taste perception among participants. This flavor advantage primarily stems from its base of concentrated grape must, which is aged in wooden barrels for several years, resulting in a layered and harmonious sweet/sour balance. The extended oxidation and interaction with wood during the aging process produce a rich profile of organic acids and volatile aromatic compounds, such as furfural, acetic acid, and esters. These compounds give balsamic vinegar a concentrated, rounded, and multi-dimensional taste impression in the mouth [39]. In contrast, the acidity of black vinegar is more straightforward, offering a clear and recognizable taste but with a relatively simple flavor profile. The acidity of black vinegar primarily comes from acetic acid produced during the fermentation of glutinous rice, with its aroma consisting mainly of small-molecule organic acids and aldehydes. Its flavor is characterized by “clean acidity, slight sweetness, and ester aroma” [14,17,40], but it lacks the complexity brought about by the aging process. The results of this study align with the findings of Olabi et al. [41], who suggested that consumers tend to show higher acceptance and preference for complex flavors when these flavors match their familiarity and expectations. Additionally, the layered flavors and sensory attributes such as viscosity, roundness, and smoothness in concentrated grape extracts can enhance consumers’ sensory evaluations [42]. This finding is also consistent with the conclusions of Wang, Niaura, and Kantono [43] and Giacalone et al. [44], who noted that multi-layered flavors in foods generally lead to higher overall evaluations. Overall, the superior performance of balsamic vinegar in the gustatory dimension is not only derived from differences in raw materials and production processes but also reflects the positive relationship between its flavor characteristics and sensory pleasure. Furthermore, Giacalone et al. [44] pointed out that when a flavor profile combines both familiarity and novelty, consumers are more likely to give positive evaluations. Balsamic vinegar embodies this advantage of balanced flavor, offering a sweet and sour taste with richness, which provides an innovative yet non-intrusive appeal to consumers accustomed to using black vinegar as a dipping sauce.

### 4.4. Sensory Evaluation of Overall Perception

The overall sensory evaluation integrated participants’ impressions and psychological responses to the samples in terms of visual, olfactory, and gustatory perceptions, as well as overall acceptance and preference. In this study, the results revealed a highly significant difference in overall sensory evaluation. Participants demonstrated significantly higher overall acceptance of balsamic vinegar compared to black vinegar, indicating that the overall advantages of balsamic vinegar in flavor consistency, aroma appeal, and mouthfeel satisfaction were successfully translated into sensory preference. This finding is consistent with Ker et al. [17], who suggested that balsamic vinegar could serve as a potential substitute for Taiwanese black vinegar. Given the combined advantages of aroma and taste, balsamic vinegar exhibited the most outstanding performance in overall sensory evaluation. This result reflects the fact that, during actual sensory evaluations, consumers tend to base their judgments on the overall flavor harmony and the pleasantness of mouthfeel rather than relying solely on a single sensory attribute. When consumers conduct an overall sensory evaluation, their assessments are generally derived from the integration of multiple sensory signals rather than being limited to one sense. When the combination of aroma and taste aligns with consumers’ familiar food memories or mental representations, these sensory cues are integrated in the brain as a unified flavor perception. This process of sensory integration enhances the harmony and recognizability of flavors, making the mixture perceived as a more enjoyable and delicious experience [45,46]. Additionally, when positive sensory experiences are generated in terms of both aroma and taste, this significantly enhances overall acceptance and preference for the product [47,48]. Overall, this study found that balsamic vinegar significantly outperformed black vinegar in overall sensory evaluation, demonstrating its comprehensive advantages in flavor, aroma, and mouthfeel. Participants’ perceptions of flavor harmony and mouthfeel pleasantness served as the primary basis for their evaluations. This suggests that overall sensory preference is derived from the integration of multiple sensory experiences rather than a judgment based on a single dimension.

### 4.5. Implications for Cross-Cultural Flavor Integration

The findings of this exploratory study suggest that consumers demonstrate openness to novel flavor profiles such as balsamic vinegar. Although this study was limited to one representative brand for each vinegar type, the consistent findings across participants provide initial insight into how Western condiments may be perceived when embedded within East Asian culinary frameworks. Future research should expand on this design by incorporating a wider variety of brands and controlled descriptors, as suggested by Torri et al. [20] and Chen et al. [22], to further validate and generalize the findings.

## 5. Conclusions

### 5.1. Conclusions

This study systematically compared black vinegar and balsamic vinegar as dipping sauces for Chinese dumplings using sensory evaluation methods, examining differences across four sensory dimensions: visual, olfactory, gustatory, and overall sensory perception. The results indicated that, except for visual evaluation, the three sample groups showed statistically significant differences in olfactory, gustatory, and overall sensory evaluations, with balsamic vinegar demonstrating the most outstanding performance. Balsamic vinegar is distinctly different from black vinegar in terms of production process, raw materials, and sensory attributes, including liquid viscosity, sweetness, and aroma. These differences highlight the potential value of balsamic vinegar as an innovative alternative to traditional black vinegar. This study not only expands the innovation of dipping sauce applications in Chinese cuisine but also illustrates the potential for cross-cultural condiments to be localized in Taiwanese cuisine. The findings provide an empirical basis for the future development of products that combine both innovation and market acceptance.

This study offers preliminary empirical evidence that Western condiments such as balsamic vinegar may be sensorially accepted in East Asian food applications. While future studies should broaden sample diversity and incorporate objective analytical instruments (e.g., electronic tongues), this work lays the groundwork for further investigations into cross-cultural sensory adaptation and product localization strategies.

### 5.2. Limitations and Future Research Directions

Although the results of this study indicate that balsamic vinegar demonstrates good sensory performance and potential acceptance as a dipping sauce for dumplings, suggesting that it could serve as an alternative to traditional black vinegar, this study is not without limitations. First, sensory preferences for condiments are highly subjective, and participants’ cultural backgrounds, dietary habits, and past experiences can significantly influence their acceptance of acidity, aroma, and mouthfeel. Second, this study employed a one-time sensory evaluation method, which assessed participants’ immediate subjective reactions. As such, it may not accurately reflect consumers’ preference stability over long-term use, actual purchase behavior, or in various consumption contexts. Third, although this study focused on the primary sensory dimensions of visual, olfactory, and gustatory perceptions, changes in taste behavior are often influenced by multiple factors, including emotions, socio-cultural context, and usage habits. Therefore, drawing conclusions solely based on sensory dimensions may have certain limitations. Fourth, this study used only one commercially available brand of black vinegar and one brand of balsamic vinegar as representatives of each type. While these brands were selected for their popularity and wide use in daily culinary practices, this narrow selection limits the generalizability of the findings across the wide variety of products available on the market. Future studies should include multiple brands to better reflect the diversity of commercially available vinegars and enhance the robustness of sensory comparison results. Moreover, it is essential to recognize that the participants in this study were primarily Taiwanese, with the majority of the sample aged between 40 and 59. This demographic focus limits the generalizability of the findings to other cultural backgrounds and population groups. Future research could include a broader range of demographic variables, combined with behavioral observation and market surveys, to gain a more comprehensive understanding of the potential application of balsamic vinegar in Chinese culinary contexts.

This study recommends that future research extend the research context to actual consumption scenarios, such as home dining, restaurant experiences, or ready-to-eat product settings, to evaluate the behavioral acceptance and repurchase intentions of using balsamic vinegar in Chinese cuisine. Additionally, the use of advanced instruments, such as electronic tongues, could be introduced for flavor analysis, providing objective data to support experimental findings from a different perspective. It is also suggested that future studies explore the cultural backgrounds of participants, conducting sensory research with cross-cultural comparisons to understand the influence of cultural factors on flavor acceptance. Furthermore, future research could adopt a health-oriented perspective, examining the differences in health perceptions and functional cognition among various types of vinegar. Lastly, it is recommended that subsequent studies integrate sensory science with a market-oriented perspective, exploring product positioning strategies and localization potential. This approach can facilitate the innovative integration and sustainable application of cross-cultural condiments in Taiwanese culinary culture.

## Figures and Tables

**Table 1:** Demographic profiles of the participants of this study.

Category	Subcategory	Frequency	Percentage (%)
Gender	Female	24	40.0
	Male	36	60.0
Age group	20 to 29 years old	9	15.0
	30 to 39 years old	8	13.3
	40 to 49 years old	17	28.3
	50 to 59 years old	19	31.7
	60 years old or above	7	11.7
Education Level	Senior High School and Vocational School	8	13.3
	University	34	56.7
	Graduate School (Including Above)	18	30.0

**Table 2:** Sensory consistency and subject representativeness analysis of AA group using independent samples *t*-test (n = 60).

Evaluation Criteria	Group	Mean (M)	Standard Deviation (SD)	df	t	*p*-Value
Visual	A1	4.26	0.49	59	−1.15	0.254
	A2	4.28	0.47			
Olfactory	A1	4.31	0.45	59	−1.65	0.103
	A2	4.34	0.43			
Gustatory	A1	4.23	0.45	59	0.70	0.487
	A2	4.21	0.45			
Overall Flavor	A1	4.17	0.46	59	−0.66	0.510
	A2	4.19	0.34			

Note: A1 = black vinegar; A2 = black vinegar.

**Table 3:** Independent samples *t*-test results for sensory evaluation of A1 and B groups (n = 60).

Evaluation Criteria	Group	Mean (M)	Standard Deviation (SD)	df	t	*p*-Value
Visual	A1	4.26	0.49	59	−1.35	0.181
	B	4.33	0.48			
Olfactory	A1	4.31	0.45	59	−2.69	0.009
	B	4.49	0.43			
Gustatory	A1	4.23	0.45	59	−2.62	0.012
	B	4.39	0.39			
Overall Flavor	A1	4.17	0.46	59	−4.27	0.000
	B	4.40	0.38			

Note: A1 = black vinegar; B = balsamic vinegar.

**Table 4:** Independent samples *t*-test results for sensory evaluation of A2 and B groups (n = 60).

Evaluation Criteria	Group	Mean (M)	Standard Deviation (SD)	df	t	*p*-Value
Visual	A2	4.28	0.47	59	−1.07	0.287
	B	4.33	0.48			
Olfactory	A2	4.34	0.43	59	−2.36	0.022
	B	4.49	0.43			
Gustatory	A2	4.21	0.45	59	−2.84	0.006
	B	4.39	0.39			
Overall Flavor	A2	4.19	0.34	59	−4.65	0.000
	B	4.40	0.38			

Note: A2 = black vinegar; B = balsamic vinegar.

**Table 5:** One-Way ANOVA Results for Sensory Evaluation of A1, A2, and B Groups (n = 60).

Evaluation Criteria		Sum of Squares	df	Mean Square	F	*p*-Value
Visual	Between Groups	0.15	2	0.08	1.53	0.221
	Within Groups	5.82	118	0.05		
	Total	6.97	120			
Olfactory	Between Groups	1.07	2	0.53	6.30	0.003
	Within Groups	9.99	118	0.09		
	Total	11.06	120			
Gustatory	Between Groups	1.12	2	0.56	7.21	0.001
	Within Groups	9.22	118	0.78		
	Total	10.34	120			
Overall Flavor	Between Groups	2.05	2	1.02	15.15	0.000
	Within Groups	7.97	118	0.07		
	Total	10.02	120			

Note: A1 = black vinegar; A2 = black vinegar; B = balsamic vinegar.

## Data Availability

Due to research ethics considerations, the data cannot be made available.
